# (-)-Epigallocatechin gallate sensitizes breast cancer cells to paclitaxel in a murine model of breast carcinoma

**DOI:** 10.1186/bcr2473

**Published:** 2010-01-15

**Authors:** Ting Luo, Jiao Wang, Yancun Yin, Hui Hua, Jing Jing, Xiangming Sun, Minjing Li, You Zhang, Yangfu Jiang

**Affiliations:** 1 State Key Laboratory of Biotherapy, Section of Signal Transduction and Molecular Targeted Therapy, West China Hospital, Sichuan University, No.1 Ke Yuan 4 Lu, Chengdu, 610041, China; 2 School of Basic Medicine, Chengdu University of Traditional Chinese Medicine, No. 17 Shi Er Qiao Lu, Chengdu, 610075, China; 3 Department of General Surgery, West China Hospital, Sichuan University, No. 37 Guo Xue Xiang, Chengdu, 610041, China

## Abstract

**Introduction:**

Paclitaxel (Taxol^®^) is a microtubule-targeted agent that is widely used for cancer treatment. However, resistance to paclitaxel is frequently encountered in the clinic. There is increasing interest in identifying compounds that may increase the sensitivity to conventional chemotherapeutic agents. In this study, we investigated whether green tea polyphenol (-)-epigallocatechin gallate (EGCG) could sensitize breast carcinoma to paclitaxel *in vivo*.

**Methods:**

Breast cancer cells were treated with or without EGCG and paclitaxel followed by detection of cell survival and apoptosis. c-Jun NH2-terminal kinase (JNK) phosphorylation and glucose-regulated protein 78 (GRP78) expression were detected by Western blotting. For *in vivo *study, 4T1 breast cancer cells were inoculated into Balb/c mice to establish a transplantation model. The tumor-bearing mice were treated with or without EGCG (30 mg/kg, i.p.) and paclitaxel (10 mg/kg, i.p.). Tumor growth was monitored. Apoptosis in tumor tissues was detected. Cell lysates from tumors were subjected to Western blot analysis of GRP78 expression and JNK phosphorylation.

**Results:**

EGCG synergistically sensitized breast cancer cells to paclitaxel *in vitro *and *in vivo*. EGCG in combination with paclitaxel significantly induced 4T1 cells apoptosis compared with each single treatment. When tumor-bearing mice were treated with paclitaxel in combination with EGCG, tumor growth was significantly inhibited, whereas the single-agent activity for paclitaxel or EGCG was poor. EGCG overcame paclitaxel-induced GRP78 expression and potentiated paclitaxel-induced JNK phosphorylation in 4T1 cells both *in vitro *and *in vivo*.

**Conclusions:**

EGCG may be used as a sensitizer to enhance the cytotoxicity of paclitaxel.

## Introduction

Paclitaxel (taxol) is a plant alkaloid commonly used in the treatment of human tumors [1]. It binds with high affinity to the β-subunit of tubulin and results in decreased dynamic instability and increased microtubule rigidity [2]. Taxol has demonstrated activity in a variety of solid tumors, particularly ovarian and breast cancer [3]. The mechanism of action of taxol is a premature stabilization of microtubule assembly, which disrupts the cytoskeletal framework necessary for tumor-cell replication and metastatic spread [4]. As a promoter of tubulin polymerization, taxol changes the dynamic equilibrium between assembly and disassembly of microtubules, disrupts the formation of the normal spindle at metaphase, and causes the blockade of mitosis at the G2/M phase [5]. Clinical practice has demonstrated that taxol plays an important role in both first-line and second-line treatment of patients with ovarian cancer and metastatic cancer of the breast [6,7]. Taxol has well-established single-agent activity in the first-line treatment of women with advanced breast cancer, with response rates for standard dose therapy ranging from 25% to 29% [8,9].

Resistance to taxol is frequently encountered in the clinic. The identification of chemosensitizers for cancer chemotherapy is an area of intensive investigation. Herbal remedies, including green tea, are emerging as popular agents for cancer patients dealing with side effects of chemotherapy. Accumulating evidence from epidemiologic, clinical and laboratory studies have revealed an inverse-relationship between increased green tea intake and the relative risk for cancer [10-12]. The chemopreventive effects of green tea have been attributed to polyphenolic ingredients that have potent antioxidant properties. Among many polyphenolic compounds isolated from green tea, (-)-epigallocatechin gallate (EGCG) is recognized as a key active constituent in terms of cancer chemopreventive potential. It is reported that 1.0 × 10^-4 ^M EGCG can significantly inhibit the growth of acute myeloblastic leukemia cells and induce apoptosis in human cancer cells [13,14]. Although cancer cell lines exhibit variable sensitivity to EGCG [15], EGCG is more and more seen as a possible new tumor-suppressing and anti-carcinogenic natural chemical. Many studies showed that EGCG inhibited the survival rate of malignant cells and induced apoptosis of malignant cells via the mitochondrial signal transduction pathway [16,17]. Roy et al. [18] reported that the increased ratio of Bax/Bcl-2 proteins after EGCG treatment might result in increased release of cytochrome C from mitochondria into cytosol, increase the expression of Apaf-1, and activate caspase-3 and poly (ADP-ribose) polymerase, which could lead to apoptosis in MDA-MB-468 cells.

An *in vitro *study demonstrated that EGCG could sensitize glioma cells to temozolomide [19]. However, EGCG reportedly blocks chemotherapy benefit of bortezomib and other boronic acid-based proteasome inhibitors. These detrimental effects of EGCG may be mediated by a direct interaction between EGCG and bortezomib thereby preventing bortezomib hitting its targets in tumor cells [20]. Therefore, it appears that EGCG can be beneficial or detrimental when it is used in combination with other agents, depending on the nature of these compounds. Many proteins have been identified as EGCG targets. One of EGCG target proteins is glucose-regulated protein 78 (GRP78), an endoplasmic reticulum (ER) chaperone that belongs to the Hsp70 family [21]. GRP78 is a key regulator of ER homeostasis due to its critical roles in protein folding, ER calcium binding, and activating transmembrane ER stress sensors [22]. As a multifunctional chaperone, GRP78 promotes tumor cell proliferation, survival, metastasis, and resistance to a variety of therapies [23]. Combination therapy suppressing GRP78 expression and activity may represent an approach toward improvement of the effectiveness of cancer therapy. Recently, we and others demonstrated that taxol could induce GRP78 overexpression, XBP-1 splicing, and eIF2α phosphorylation, hallmarks of the unfolded protein response [24,25]. GRP78 knockdown potentiates taxol-induced JNK phosphorylation and protects breast cancer cells against paclitaxel-induced apoptosis [24]. In this regard, GRP78 may be a novel target to overcome taxol resistance. Based on these earlier findings, we initiated this study to investigate whether EGCG is able to enhance the anti-tumor effects of taxol *in vivo*.

## Materials and methods

### Reagents

Paclitaxel (Taxol) were purchased from Sigma-Aldrich, Inc. (St. Louis, MO, USA). Ninety-nine percent pure EGCG was purchased from MUST Biotech. (Chengdu, China). The phosphorylated JNK antibody and JNK inhibitor SP600125 were provided by Cell Signaling (Beverly, MA, USA). GRP78 and β-actin antibodies were purchased from Santa Cruz Biotechnology (Santa Cruz, CA, USA).

### Cell culture

Breast cancer cell lines (4T1, MCF-7, and MDA-MB-231) were originally obtained from the American Tissue Culture Collection. Tumor cells were grown in tissue culture flasks at 37°C in a humidified atmosphere of 5% CO_2 _and were maintained as monolayer cultures in Dulbecco's minimal essential medium supplemented with 5% fetal bovine serum, 100 U/mL penicillin and 100 μg/mL streptomycin.

### 4-[3-(4-iodophenyl)-2-(4-nitrophenyl)-2H-5-tetrazolio]-1,3-benzene disulfonate (WST-1) assay

Cells were plated in 96-well plates at 5,000 cells per well. The next day, cells were treated with or without taxol and 20 μM EGCG in four replicates. After 24 or 48 h, cell viability was assessed by incubating cells with WST-1 reagent (Roche, Indianapolis, IN, USA) for 2 h and measuring the absorbance at 450 nm, and at 630 nm as reference, with a microplate reader (Bio-Rad, Hercules, CA, USA).

### Hoechst 33342 staining

Replicate cultures of 1 × 10^6 ^cells per well were plated in 24-well plate. The cells were treated with or without 20 μM EGCG, 1 μM taxol and 10 μM SP600125. After a change of fresh medium 24 h later, the cells were incubated with 5 μL of Hoechst 33342 solution per well at 37°C for 10 minutes, followed by observation under a fluorescence microscope. Strong fluorescence can be observed in the nuclei of apoptotic cells, while weak fluorescence was observed in non-apoptotic cells. Quantification of apoptotic cells was performed by counting cells in four random fields in each well.

### Western blot

For cell cultures, cells were treated with 1 μM taxol, 20 μM EGCG, or combination for 24 h. The cells were washed twice with phosphate buffered saline and harvested with cold lysis buffer containing protease inhibitors or phosphatase inhibitors. Cell lysates were collected from culture plates using a rubber policeman, and protein collected by centrifugation. For tumor samples, tumor tissues were homogenized in lysis buffer containing protease inhibitors, and protein collected by centrifugation. Protein concentrations were determined by BCA protein assay (Pierce Biotechnology, Rockford, IL, USA). Forty micrograms of total protein were boiled in 2× loading buffer (0.1 M Tris-Cl, pH 6.8, 4% SDS, 0.2% bromophenyl blue, 20% glycerol) for 10 minutes, then loaded into Tris-HCl-Polyacrylamide gels, and transferred electrophoretically to Immobilon-P membrane (Millipore Corporation, Billerica, MA, USA). Membranes were incubated with primary antibodies and appropriate horseradish peroxidase-labeled secondary antibodies. Membranes were additionally probed with an antibody against β-actin (Santa Cruz Biotechnology) to normalize loading of protein among samples. The secondary antibodies were detected by chemiluminescent agents (Pierce Biotechnology).

### Determination of tumor inhibition in a murine model of mammary carcinoma

All animal studies were performed according to the guidelines and approval of the Institutional Review Board of West China Hospital. Six- to seven-week-old female Balb/c mice were used for all experiments. The mice were housed in groups of four to five animals per cage. To establish tumor grafts, subconfluent breast cancer cells 4T1 were dispersed with 0.1% trypsin/EDTA and washed once with medium containing 5% calf serum to remove the trypsin. The cells were resuspended at a concentration of 8 × 10^5 ^cells/mL in phosphate-buffered saline. A total of 8 × 10^4 ^breast cancer cells were injected subcutaneously and permitted to grow until palpable. At that time, the mice were randomly assigned into control and treatment groups and chemotherapy was initiated. The doses and route of administration for EGCG and taxol were chosen according to reports by Scandlyn et al [26] and Wang et al [27], respectively. EGCG (30 mg/kg) was delivered intraperitoneally everyday, while taxol (10 mg/kg) was given intraperitoneally every two days. Control animals received an injection of 0.9% saline solution in volumes equivalent to those used for injection of the drugs.

Two-dimensional measurements were taken with calipers during the treatment period, and tumor volume was calculated with the use of the following formula: tumor volume (mm^3^) = a × b^2 ^× 0.52, where *a *is the longest diameter, *b *is the shortest diameter. At the end of the experiments, the mice were sacrificed by carbon dioxide aspiration, the tumors were dissected, fixed in formalin, and embedded in paraffin. Visible metastatic foci in the lungs were counted.

### DNA fragmentation detection

Cell apoptosis in tumor tissues was analyzed using the Fluorescein-FragEL™ DNA Fragmentation Detection Kit (Calbiochem, San Diego, CA, USA) according to the manufacturer's instruction. The apoptotic index was evaluated by the percentage of cells scored under a light microscope at 200-fold magnification.

### Statistical analysis

A two-way repeated measures ANOVA was used to test for the differences in tumor growth. One-way ANOVA was used to test for the difference in the means of apoptosis rate, tumor weight, and metastasis. All statistical tests were two-tailed, and difference to be considered to be statistically significant when *P *< 0.05.

## Results

### EGCG sensitizes breast cancer cells to taxol-induced apoptosis

**Figure 1:**
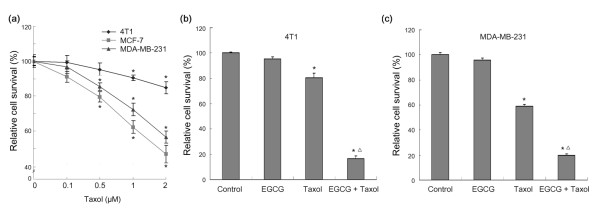
**EGCG sensitizes breast cancer cells to taxol**. **(a) **Sensitivity of 4T1, MDA-MB-231 and MCF-7 cells to taxol. Cells were treated with increasing doses of taxol (0.1 to 2 μM) for 24 h. Cell viability was assessed by WST1 assay. *Points *= mean of four replicates; *bars *= SE. A*sterisk *= *P *< 0.01 versus untreated controls. **(b) **4T1 cells were seeded in a 96-well plate at 5,000 cells per well. The next day, the cells were treated with 20 μM EGCG, 1 μM taxol, or a combination for 48 h. Cell viability was assessed by WST1 assay. *Points *= mean of four replicates; *bars *= SE. *Asterisk = P *< 0.01 versus untreated control. *Triangle = P *< 0.001 versus single agent. **(c) **MDA-MB-231 cells were seeded in a 96-well plate at 5,000 cells per well. The next day, the cells were treated with 20 μM EGCG, 1 μM taxol, or combination for 48 h. Cell viability was assessed by WST1 assay. *Points *= mean of four replicates; *bars *= SE. *Asterisk *= *P *< 0.001 versus untreated control. *Triangle *= *P *< 0.001 versus single agent.

**Figure 2:**
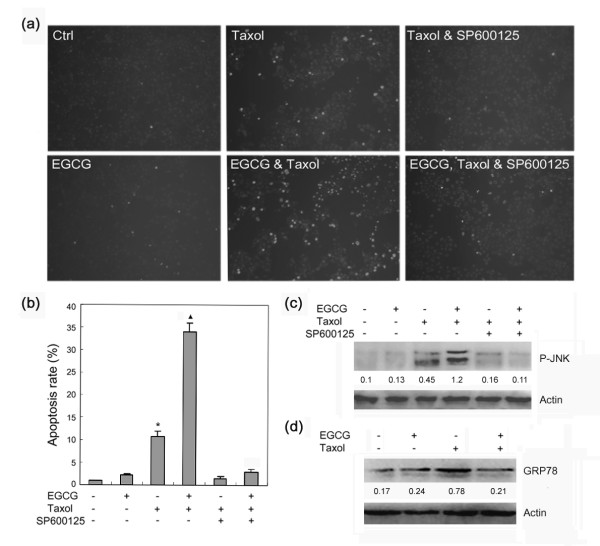
**EGCG potentiates taxol-induced apoptosis in 4T1 cells**. **(a) **4T1 cells were treated with 20 μM EGCG, 1 μM taxol, 10 μM SP600125 or combination for 24 h, and apoptosis was assessed by Hoechst 33342 staining. The apoptotic cells with strong fluorescence or fragmented nuclei were observed under fluorescent microscopy. **(b) **Quantification of apoptotic cells was performed by taking the images in random fields and counting cells with strong fluoresence or fragmented nuclei. The relative apoptosis rate was plotted. *Columns *= mean percentage of apoptotic cells; *bars *= SE. *Asterisk = P *< 0.001 versus untreated control or taxol in combination with 10 μM SP600125. *Black triangle = P *< 0.001 versus EGCG, taxol, or taxol in combination with EGCG and SP600125. **(c) **4T1 cells were treated with 20 μM EGCG, 1 μM taxol, EGCG in combination with taxol, taxol in combination with 10 μM SP600125, or taxol in combination with EGCG and SP600125 for 24 h. Total proteins were harvested with lysis buffer containing phosphatase inhibitor, and subjected to Western blot analysis of JNK phosphorylation. The ratio of p-JNK/actin as determined by densitometric analysis was given. **(d) **4T1 cells were treated with 20 μM EGCG, 1 μM taxol, or EGCG in combination with taxol for 24 h. Total proteins were harvested and subjected to Western blot analysis of GRP78 expression. The ratio of GRP78/actin as determined by densitometric analysis was given. Representative of two experiments was shown.

First, we determined the sensitivity of three breast cancer cell lines (4T1, MCF-7, and MDA-MB-231) to taxol. Among three cell lines, 4T1 was less sensitive to taxol than MCF-7 and MDA-MB-231 (Figure 1a). Next, the effect of EGCG, taxol and a combination of the two on cell survival was examined by WST1 assay. Treatment of 4T1 and MDA-MB-231 cells with EGCG in combination with taxol led to a dramatic decrease in cell viability compared to treatment with taxol alone (Figure 1b and 1c). To determine whether the combination of EGCG and taxol can synergistically promote apoptosis in 4T1 cells, the effect of EGCG, taxol and combination on apoptosis was examined by Hoechst 33342 staining. The results demonstrated that treatment of 4T1 cells with EGCG in combination with taxol led to a dramatic increase in cell apoptosis compared to treatment with taxol alone (Figure 2a and 2b).

Previous studies have demonstrated that JNK activation is involved in taxol-induced apoptosis. Here, we found that JNK inhibitor also suppressed the stimulatory effects of EGCG on taxol-induced apoptosis in 4T1 cells (Figure 2a and 2b). To determine whether EGCG indeed affect taxol-induced JNK phosphorylation in 4T1 cells, the cells were treated with EGCG, taxol, or both. Treatment with EGCG alone did not induce JNK phosphorylation. However, a combination of EGCG and taxol resulted in higher levels of phosphorylated JNK than that in cells treated with taxol alone (Figure 2c). Treatment with SP600125 reduced the levels of phosphoralated JNK. These results demonstrated that EGCG could potentiate the activation of JNK by taxol.

Previously, it has been demonstrated that paclitaxel and docetaxel can induce GRP78 expression [22,23]. GRP78 knockdown potentiated taxol-induced JNK phosphorylation and cell apoptosis [22]. To determine the effect of EGCG on taxol-induced GRP78 expression, we checked the levels of GRP78 in 4T1 cells treated with EGCG, taxol, or both. The result demonstrated that EGCG suppressed taxol-induced GRP78 expression (Figure 2d).

### EGCG sensitizes breast carcinoma to taxol *in vivo*

**Figure 3:**
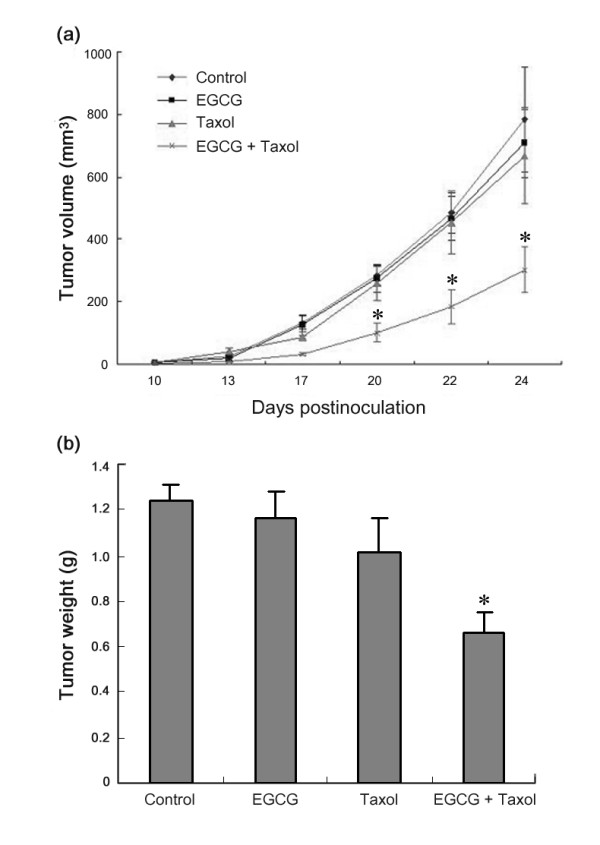
**EGCG sensitizes 4T1 tumors to taxol in Balb/c mice**. **(a) **4T1 cells were injected into Balb/c mice as described in Materials and Methods. When tumors became palpable, the mice were randomly assigned into different groups (n = 8 for control; n = 8 for EGCG group; n = 8 for taxol group; n = 10 for combination treatment), and treated with EGCG (30 mg/kg), taxol (10 mg/kg), or both. Tumor growth was monitored for 24 days. *Asterisk = P *< 0.05, significantly different from control, EGCG, or taxol group (two-way ANOVA, *post-hoc *test). Data are mean ± SE. **(b) **Tumors were dissected 24 days after inoculation of tumor cells into the mice. The tumor weight was measured. The combined data of tumor weight from two experiments was plotted (n = 15 for control; n = 15 for EGCG group; n = 15 for taxol group; n = 16 for combination treatment). *Columns *= mean of tumor weight; *bars *= SE. *Asterisk *= *P *< 0.05, significantly different from control or single agent.

To determine whether EGCG can improve the therapeutic effects of taxol on breast carcinoma *in vivo*, the effect of combination treatment with EGCG and taxol on mammary tumorigenesis was evaluated in a murine breast cancer (4T1) model in Balb/c mice. When tumors were palpable, therapy with EGCG was initiated at 30 mg/kg body weight everyday, and therapy with taxol was initiated at 10 mg/kg body weight every two days. Combination treatment with EGCG and taxol was also initiated. Control mice received 0.9% saline solution. Tumors were not sensitive to taxol at the given dose. Whereas taxol or EGCG alone had little effect on tumor growth, the combination of EGCG and taxol significantly inhibited tumor growth (Figure 3). These data demonstrated that EGCG could sensitize 4T1 tumors to taxol *in vivo.*

In addition, we examined the effect of combination treatment with EGCG and taxol on lung metastasis. Twenty-four days after inoculation of 4T1 cells in Balb/c mice, the tiny metastatic foci in the lung surface were observed. After counting visible metastasis, we found that the median of metastasis by treatment was as follows: control, 5 (95% confidence interval (CI), 0 to 15.7); EGCG, 3; (95% CI, 0 to 5.7); taxol, 7.5 (95% CI, 0 to 16.9); and EGCG plus taxol, 2 (95% CI = 0 to 3.9). There was no significant difference between the control group and taxol- or EGCG-treated group, while the difference between the control group and combination treatment group was marginally significant (*P *= 0.053).

Animals treated with EGCG and taxol had no significant changes in weight (data not shown), suggesting no overt systemic toxicity. In addition, systematic examination of major organs revealed no histological changes indicative of drug toxicity, including liver, spleen, heart, and kidneys.

### EGCG promotes taxol-induced apoptosis and overcomes taxol-induced GRP78 expression in tumor tissues

**Figure 4:**
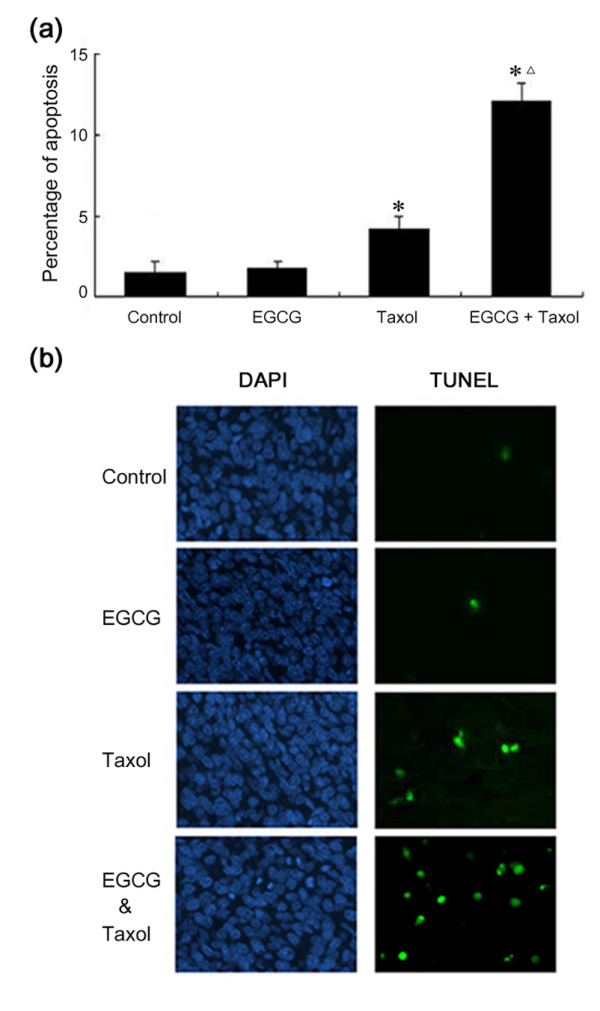
**EGCG potentiates taxol-induced apoptosis in tumors**. **(a) **Percent apoptosis relative to the saline-treated control tumors. *Columns *= mean of apoptosis rate in tumors; *bars *= SE. *Asterisk = P *< 0.01, significantly different from control. *Triangle = P *< 0.001, significantly different from taxol alone. **(b) **Representative TUNEL assays for tumors were shown.

We detected the apoptosis indices in tumor tissue by *in situ *DNA fragmentation assay. The control tumors had an average apoptosis index of 1.5%. The EGCG-treated tumors had an average apoptosis index of 1.8%. The taxol-treated tumors had an average apoptosis index of 4.2%. The tumors that were treated with both EGCG and taxol had an average apoptosis index of 12.1% (Figure 4). In addition, we determined the proliferation index of tumor cells by immunostaining tumor sections for proliferating cell nuclear antigen, a nuclear marker for proliferative cells. There was no significant difference in the proliferation indices among these groups of tumors (data not shown).

**Figure 5:**
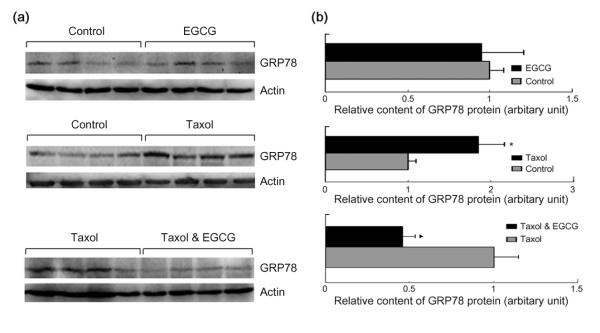
**EGCG overcomes taxol-induced GRP78 expression in tumors**. Cell lysates from dissected tumor tissues were subjected to Western blot analysis of GRP78 expression. **(a) **The levels of GRP78 protein in tumors treated with vehicle, EGCG, and taxol were shown. **(b) **Quantitative analysis of GRP78 expression after normalization to actin by densitometric analysis. The ratio of GRP78/actin represented by grey column was set as 1 arbitary unit. *Columns *= mean of relative levels of GRP78 protein; *bars *= SE. *Asterisk *= *P *< 0.05 versus control. *Black triangle *= *P *< 0.05 versus taxol.

Previously, our *in vitro *study demonstrated that taxol up-regulated the expression of the endoplasmic reticulum chaperone GRP78, one of EGCG targets. To determine whether EGCG and taxol affect GRP78 expression in tumor tissues, we detected GRP78 levels in tumors by Western blotting. Overall, the levels of GRP78 protein tend to be increased in taxol-treated tumors (Figure 5). The levels of GRP78 in tumors treated with both EGCG and taxol were lower than that in taxol-treated tumors, suggesting that EGCG could overcome taxol-induced GRP78 expression. These data confirmed that taxol induced GRP78 expression *in vivo. *Since GRP78 confers taxol resistance, this study validated GRP78 as a target for overcoming taxol resistance.

**Figure 6:**
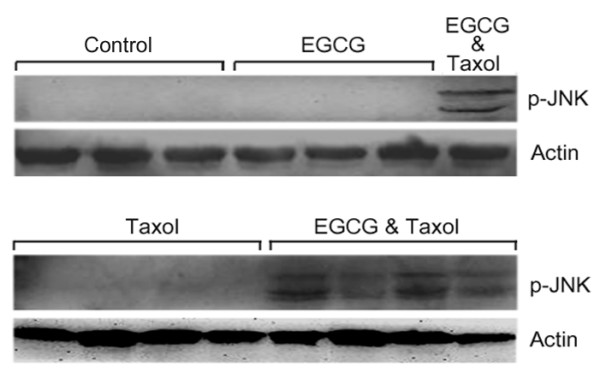
**EGCG potentiates taxol-induced JNK phosphorylation in tumors**. Cell lysates from dissected tumors were subjected to Western blot analysis of JNK phosphorylation. The levels of phosphorylated JNK in tumors treated with or without EGCG and taxol were shown.

In addition, we investigated JNK phosphorylation in tumors that were treated with or without EGCG and taxol. EGCG in combination with taxol markedly induced JNK phosphorylation in tumor tissues, whereas phosphorylated JNK was barely detected in tumors treated with taxol or EGCG alone (Figure 6).

## Discussion

Taxol has been used extensively to treat lung, ovarian and breast cancers but drug resistance limits the clinical usefulness of this drug. Previous studies have disclosed some mechanisms underlying taxol resistance. Due to its hydrophobic nature, resistance to taxol is associated with the induction of the multidrug resistance gene (MDR-1) encoding P-glycoprotein [28] and a decreased cellular accumulation of taxol [29,30]. Moreover, previous studies demonstrated that several markers might be associated with taxol sensitivity/resistance, including p53, BRCA2, members of the epidermal growth factor receptor (EGFR) superfamily (for example, HER2, EGFR), estrogen receptors and progesterone receptors [31-35]. However, retrospective clinicopathologic study found that none of these molecular markers were predictive of response to single-agent taxane chemotherapy in patients with metastatic breast cancer [36]. We and others have previously found that taxol could induce the unfolded protein response [24,25]. GRP78 is a multifunctional protein involved in the unfolded protein response, chemoresistance, cell proliferation and angiogenesis. Abrogation of GRP78 induction sensitizes breast cancer cells to taxol by potentiating the activation of pro-apoptotic signal such as JNK phosphorylation and caspase cleavage.

Identification of sensitizers for taxol is expected to improve the efficacy of taxol in treating human tumors. 17-Allylamino-17-demethoxygeldanamycin (17-AAG) inhibits Hsp90, a molecular chaperone that is important for the posttranslational folding and stability of some kinases, which are considered important targets for cancer therapy. 17-AAG, by virtue of its effects on multiple signal transduction pathways, may therefore overcome resistance to molecular targeted therapy. Also, previous studies suggested that there may be additional potential for its use in combination with conventional agents because some of the pathways inhibited by 17-AAG are also implicated in cytotoxic drug resistance [37]. 17-AAG may sensitize a subset of ovarian cancer to paclitaxel, particularly those tumors in which resistance is driven by Hsp90 clients such as ERBB2 and/or p-Akt [38]. Since GRP78 is involved in taxol response, it may be another heat shock protein that can be targeted to improve the efficacy of taxol.

The use of a non-toxic modulator to enhance the antitumor activity is invaluable in improving the outcome of patients with cancer. Green tea has been reported to have useful pharmacological effects such as inhibition of carcinogenesis, chemoprevention of cancer, and antioxidative effect [39-42]. EGCG and flavonoids have inhibitory effects on the doxorubicin efflux from Ehrlich ascites carcinoma cells [43]. Oral administration of green tea can enhance the inhibitory effects of doxorubicin on Ehrlich ascites carcinoma in tumor-bearing mice [44]. The combination of EGCG and curcumin suppressed tumor growth in a mouse model of human breast carcinoma, which correlated with a significant decrease in levels of VEGFR-1 in the tumors [45]. In that study, tumor growth in a combination-treated group was inhibited by 49%, while the tumor-suppressing rate in an EGCG-treated group was 31% [35]. Co-administration of EGCG and tamoxifen synergistically suppressed tumor growth in a mouse model of human estrogen receptor negative breast cancer (MDA-MB-231) [26]. The tumor-suppressing rate for EGCG in combination with tamoxifen reached 80%, while EGCG alone inhibited tumor growth by 35% [26]. EGCG also synergistically enhanced the inhibitory effects of sulindac or tamoxifen on human lung cancer cells [46]. In our study, when given at a similar dose, EGCG alone did not inhibit 4T1 breast tumor growth. EGCG in combination with taxol resulted in a decrease in tumor weight by 47%, whereas taxol alone inhibited tumor growth by 22.5%. In addition, the lung metastasis in mice treated with EGCG in combination with taxol was less than that in mice treated with vehicle or taxol alone, while this difference was marginally significant, which might be due to the variance in control mice. The effects of EGCG on tumor progression may vary among different cell types or cell lines. Also, the tumor-suppressive effects of EGCG in combination with other agents may depend on the nature of these compounds.

The mechanisms underlying the anticancer effects of EGCG seem to be complex. EGCG can bind to target molecules and trigger signaling cascades, many of which are interconnected. It has been reported that EGCG may bind to IGF-1R [47], GRP78 [21], Hsp90 [48], and BCL-2 [49]. As a GRP78 inhibitor, EGCG reportedly overcame resistance to etoposide-induced cell death *in vitro *[21]. The current study demonstrates that EGCG could sensitize breast carcinoma to taxol in a murine model. Notably, EGCG can overcome taxol-induced GRP78 expression, which represents another mechanism underlying EGCG suppression of GRP78. Activation of JNK plays critical roles in taxol-induced apoptosis. GRP78 knockdown potentiates taxol-induced JNK phosphorylation [24]. Co-treatment with EGCG potentiates the activation of JNK in cancer cells by taxol both *in vitro *and *in vivo*. Cell apoptosis in breast cancer grafts is enhanced by combination of EGCG and taxol, resulting in decreased tumor burden. Since EGCG can target multiple molecules that are involved in cell survival, one can speculate that the molecular events that are responsible for EGCG potentiation of taxol-induced apoptosis may involve more than one target. Non-apoptotic cell death may also be involved in growth inhibition observed in this study. Given that taxol upregulates GRP78 expression and that GRP78 knockdown sensitizes breast cancer cells to taxol [24], it is reasonable to suggest that inhibition of GRP78 may mediate, at least in part, the enhancement of taxol sensitivity by EGCG.

Bioavailability is an important issue with natural chemicals such as polyphenolic compounds. In this regard, a prodrug of EGCG has been developed to increase the stability, bioavailability and anticancer activities of EGCG [50]. In addition, the bioavailability and effectiveness of EGCG can be improved by encapsulating it in nanoparticles [51]. The development of other bioavailable and stable EGCG may greatly improve its utility in cancer prevention and therapeutics.

## Conclusions

This study demonstrated that treatment with EGCG significantly increased paclitaxel-induced JNK phosphorylation and cell death, and improved the efficacy of paclitaxel therapy *in vivo*. Thus, we present an alternative approach for increasing breast cancer cell sensitivity to paclitaxel. The efficacy of the EGCG-paclitaxel combination in patients with breast cancer or other solid tumors warrants further study.

## Abbreviations

17-AAG: 17-Allylamino-17-demethoxygeldanamycin; EGCG: (-)-epigallocatechin gallate; ER: endoplasmic reticulum; GRP: glucose-regulated protein; HSP: heat shock protein; JNK: c-Jun NH2-terminal kinase; TUNEL: Terminal deoxynucleotidyl transferase (TdT)-mediated dUTP nick end labeling; WST-1: 4-[3-(4-iodophenyl)-2-(4-nitrophenyl)-2H-5-tetrazolio]-1,3-benzene disulfonate.

## Competing interests

The authors declare that they have no competing interests.

## Authors' contributions

YJ participated in designing the experiments and in analyzing the data and wrote the manuscript. TL participated in carrying out cell culture, animal studies and in analyzing the data. JW participated in carrying out animal studies, Hoechst 33342 staining and DNA fragmentation detection. YY participated in carrying out WST1 assay. HH and JJ participated in carrying out Western blotting. XS, ML, YZ assisted in collecting samples from tumor-bearing mice, embedding tissues in paraffin. All authors read and approved the final manuscript.
